# Attempts at Soft and Hard Tissue Augmentations During Surgically Facilitated Orthodontic Therapy

**DOI:** 10.3390/dj14060335

**Published:** 2026-06-02

**Authors:** Jason Poon, Tun-Jan Wang, Nipul Tanna, Min Yang, Anas Baghareeb, Chun-Hsi Chung, Yu-Cheng Chang, Chenshuang Li

**Affiliations:** 1 Department of Orthodontics, School of Dental Medicine, University of Pennsylvania, Philadelphia, PA 19104, USA; jdpoon22@upenn.edu (J.P.); nipul77@upenn.edu (N.T.); sherryy@bu.edu (M.Y.); baghareb@upenn.edu (A.B.); chunc@upenn.edu (C.-H.C.); 2 Department of Periodontics, School of Dental Medicine, University of Pennsylvania, Philadelphia, PA 19104, USA; tjwang@upenn.edu

**Keywords:** Phenotype, periodontics, orthodontics, collagen matrix

## Abstract

**Background:** Over the past two decades, with the collaboration between periodontists and orthodontists, the concept of a surgically facilitated orthodontic therapy (SFOT) approach was developed, which not only provides acceleration in tooth movement as a result of a stimulated regional acceleratory phenomenon but also increases the alveolar bone volume for orthodontic tooth movement range by bone grafting. Despite the benefits, hard and soft tissue defects can sometimes be observed in patients post SFOT, especially among groups with thin periodontal phenotypes. **Methods:** In the current study, we compared the traditional SFOT with allograft only and the modified SFOT with allograft and collagen matrix to explore if the addition of a collagen matrix between the bone graft and periodontal flap could achieve soft tissue augmentation and reduce the prevalence of the complications of SFOT. Six patients were initially enrolled in the current study. Four patients showed up for the 1-week and 2-week post-operation evaluations. However, only one subject of each group agreed on periodontal evaluation by bone sounding and CBCT during the 6-month follow-up appointment. **Results:** Six months post SFOT, the patient with the modified SFOT presented with a larger amount of soft tissue thickening compared to the patient with traditional SFOT. However, the location of bone generation was different between groups, with the modified SFOT group presenting with bone generation more apically. In addition, patients with modified SFOT reported a more severe level of discomfort than patients with traditional SFOT. **Conclusions:** The modified SFOT may potentially improve soft tissue phenotype; however, further modifications are needed to reduce the patient’s discomfort level and to ensure that bone particles are contained more incisally.

## 1. Introduction

The orthodontic tooth movements strongly rely on the interactions between the teeth and their supportive periodontal tissues [1], with the pre-existing alveolar volume boundary limiting the range of orthodontic tooth movement. The expectation for high-quality orthodontic treatment with a shorter treatment time and maintenance of periodontal health is increasing in our society. In response to this demand, and through collaboration between periodontists and orthodontists [2], the concept of the surgically facilitated orthodontic therapy (SFOT) approach has been developed, which provides acceleration in tooth movement as a result of a stimulated regional acceleratory phenomenon [3] and expands the pre-existing alveolar volume boundary for orthodontic tooth movement [4]. In its traditional acceleratory application, SFOT/PAOO combines selective corticotomy with particulate grafting in dentoalveolar sites where activated, vascularized alveolar bone and periosteal/flap blood supply are expected to support accelerated orthodontic tooth movement and maintenance of the dentoalveolar envelope [5,6].

Hard and soft tissue defects, such as dehiscence, fenestration, and recession, can sometimes be observed in patients with thin periodontal phenotypes [7]. These defects can affect periodontal health and tooth prognosis over time [8]. In fact, patients with thin scalloped phenotypes have been associated with a compromised soft tissue response following surgical and restorative treatment [9]. This may be attributed to less blood supply nourishing the underlying bone [10], where thinner phenotypes may present with more dramatic alveolar resorption after tooth extraction or gingival inflammation [11], and even reduced implant success [12]. Thus, Beauchamp et al. emphasized that SFOT should aim to enhance and thicken both hard and soft tissue [13]. This concept is supported by the American Academy of Periodontology’s (AAP) best evidence review on CBCT analysis on identifying patients with higher risk for periodontal–orthodontic compromise, which reported that augmented corticotomy combined with accelerated orthodontic forces increased buccal alveolar bone thickness, helped prevent the development of bony dehiscences, and did not result in significant crestal bone loss when evaluated with CBCT imaging [14]. Accordingly, SFOT/PAOO may be considered not only as an acceleratory orthodontic procedure, but also as a dentoalveolar augmentation approach for anatomically vulnerable patients with thin periodontal phenotypes, dehiscence/fenestration defects, or planned tooth movement beyond the existing alveolar housing. In this setting, the clinical intent is not to claim periodontal regeneration on avascular root surfaces, but rather to augment the buccal dentoalveolar envelope and reduce future root exposure. This distinction is important because true periodontal regeneration requires histologic confirmation of a new cementum, periodontal ligament, and alveolar bone. Despite the histological limitation, end points for augmentation assessments, according to the American Academy of Periodontology’s best evidence review on phenotype modification, otherwise require notations on probing depths, gingival recession, keratinized tissue width, and CBCT or lateral cephalometric radiographic assessment of bone thickness, whether due to newly generated bone, residual graft material, or an incorporation of both after augmentation [6].

Conversion of the patient’s soft tissue phenotype both quantitatively and qualitatively through soft tissue augmentation, such as free gingival grafting or connective tissue grafting, provides more predictable surgical and prosthetic outcomes. However, free gingival grafting not only requires a secondary surgical site for graft harvesting, which increases patients’ discomfort and surgical morbidity, but it is also limited by the availability of donor tissue. Thus, developing a modified SFOT protocol that combines different grafting materials is warranted to further improve the combined periodontal–orthodontic care, especially for patients with thin phenotypes. Collagen matrix, a porcine-derived, resorbable, and volume-stable graft material, has been used in periodontal surgeries for soft tissue augmentation around natural teeth and implants. Multiple studies included in two systematic reviews [15,16] have demonstrated that the use of a collagen matrix could achieve similar soft tissue volume to that of autogenous grafts while eliminating the need for a second surgical site, reducing procedure time, and delivering greater treatment flexibility. In addition, using a membrane during bone augmentation procedures could help achieve wound stability and space maintenance [3]. Stern et al. also suggested that a membrane is beneficial and recommended that its use be incorporated into SFOT protocols [3]. Accordingly, in the present protocol, the particulate graft was intended to provide buccal contour support and space maintenance, while wound healing was expected to depend primarily on vascular and cellular contributions from adjacent alveolar bone, the remaining periodontal ligament at the defect margins, periosteal/supraperiosteal circulation, and the overlying flap, rather than from the denuded root surface itself [6,17]. The collagen matrix was added as a soft tissue phenotype-modifying layer between the bone graft and periodontal flap; however, its use in sites with extensive root exposure and limited native bony walls remains biologically and surgically demanding. Thus, we conducted the clinical procedure to evaluate whether the addition of a collagen matrix between the bone graft and periodontal flap during corticotomy surgery could enhance the soft tissue phenotype, improve buccal hard-tissue augmentation, and in turn, reduce the prevalence of SFOT-related complications in patients.

## 2. Materials and Methods

### 2.1. Subjects

As a proof-of-concept interventional study, the current study only included adult patients who were systemically (American Society of Anesthesiologists (ASA) Physical Status Classification I and II) and periodontally healthy, non-smokers, who needed orthodontic treatment, who could benefit from a periodontal accelerated orthodontic treatment on the labial side of the mandibular arch, and who voluntarily signed the informed consent form. The patients each presented with required tooth movement, wherein final tooth positions would place roots beyond the existing alveolar housing. The surgery was planned at the time when a significant amount of labial movement would be expected, such as (1) increased labial root torque from upgrading the arch wire to rectangular stainless steel wires; (2) proclined lower incisors from the arch leveling and aligning; or (3) mesialization of the mandibular dentition from the usage of class II elastics. The patients were treated with either clear aligners or a fixed labial bracket system, per the patients’ preferences. This study was approved by the institution review board (protocol # 850969, approved on 23 May 2023).

Six patients were initially enrolled in the current study, with three patients in the group treated with the traditional SFOT technique (bone particles only), and three subjects in the group treated with the modified protocol of SFOT with the combination of collagen matrix and bone particles. Five patients performed the SFOT procedure as planned. Four patients showed up for the 1-week and 2-week post-operation evaluations. However, only three patients showed up for both the 1-month and 6-month follow-up appointments, and only one subject from each group agreed to undergo periodontal evaluation by bone sounding and CBCT during the 6-month follow-up appointment (Table 1).

### 2.2. Surgical Techniques

**Figure 1:**
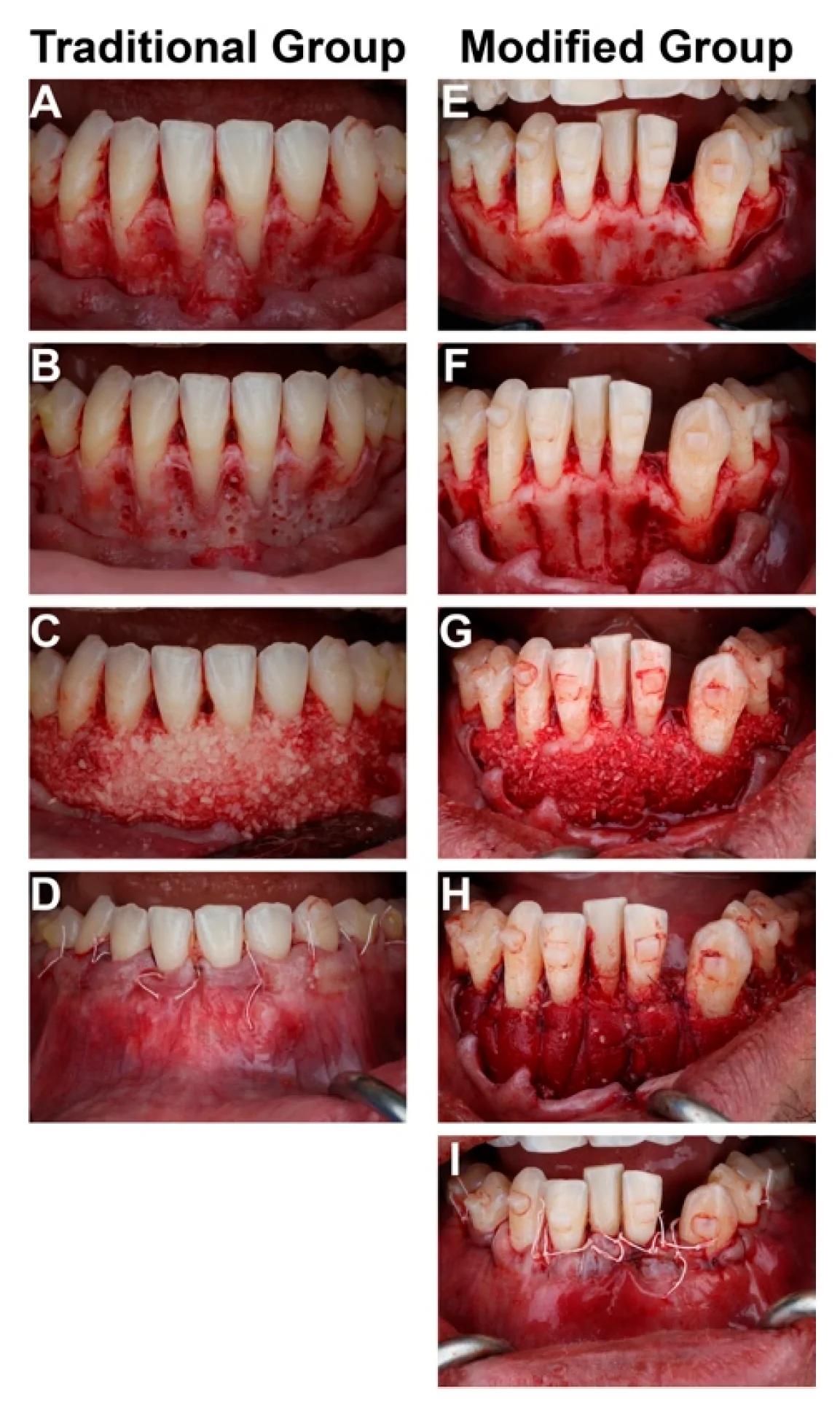
The step-by-step surgical photos of the traditional (**A**–**D**) and modified (**E**–**I**) SFOT.

For the traditional SFOT group, a full-thickness flap was raised from the mesial papilla of the mandibular first molar to the contralateral mandibular first molar, followed by tunneling beyond the distal line angle. The flap was reflected apically 15 mm from the CEJ (Figure 1A). Bone activation was performed with columnar corticotomies and intramarrow penetrations (Figure 1B). Allograft bone graft material (Puros^®^ Cortico-Cancellous Particulate, Zimmer Biomet, Warsaw, IN, USA) was placed on the buccal aspect of any dehiscences, fenestrations, and native alveolar bone (Figure 1C). The flap was repositioned and sutured with 4-0 polytetrafluoroethylene sutures (Salvin CytoSurg^®^ PTFE 4-0 P-3, Salvin Dental Specialties, LLC, Charlotte, NC, USA) (Figure 1D).

For the modified SFOT group, this group had the same flap design, bone activation, and allograft bone graft material as the traditional SFOT group (Figure 1E–G). The bone particulate was then covered with a 1.5 mm thick collagen matrix (Geistlich Fibro-Gide^®^, Geistlich Pharma North America, Inc., Princeton, NJ, USA) (Figure 1H). Periosteal releasing incisions were completed to release tension from the flap and then the collagen matrix was secured in place with internal sling 4-0 Glycolon sutures (Resorba^®^ Glycolon™ Absorbable Suture, Osteogenics Biomedical, Lubbock, TX, USA). The flap was then repositioned and sutured as described in the traditional SFOT group (Figure 1I).

All surgical procedures were performed by the same periodontist.

### 2.3. Postoperative Care

All participants were administered 500 mg of amoxicillin for 7 days postoperatively, and were instructed to gently rinse with warm salt water twice a day. Pain management consisted of a combination of ibuprofen and acetaminophen. The modified SFOT group was prescribed methylprednisolone to decrease swelling. Patients were instructed to avoid pressure over the surgical site and avoid brushing in the grafting area for 2 weeks.

### 2.4. Adverse Event Collection

**Figure 2:**
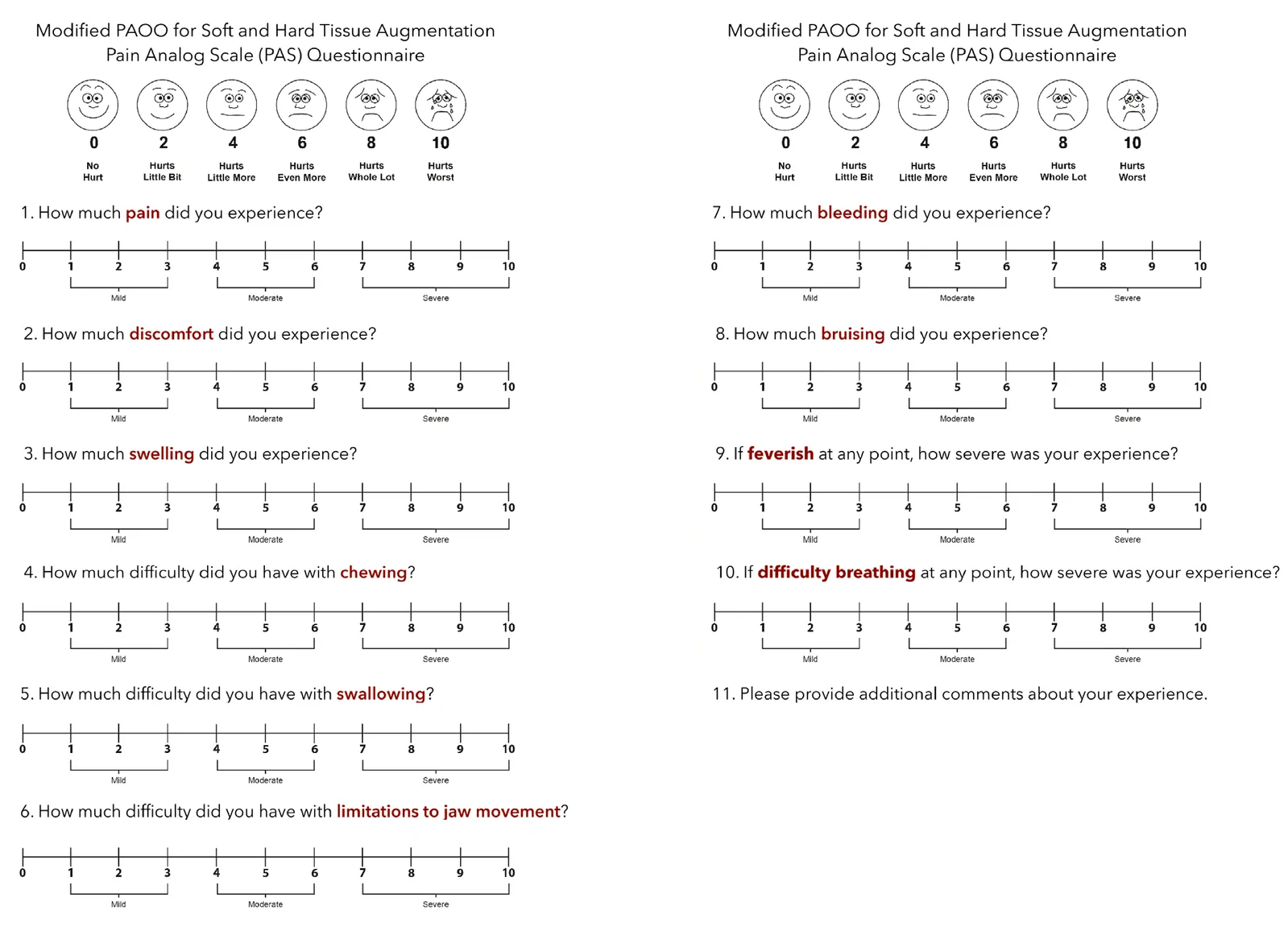
The pain analog scale questionnaire utilized in the current study.

One week and two weeks after the operation, the patients revisited the clinic and were evaluated again by the periodontist to document any adverse event, swelling, and perceived pain. Quantification and measurement of pain were measured using a 10 cm horizontal visual analog scale (VAS) where 0 represents no pain and 10 represents the most severe pain, and the need for post-operative analgesics [18,19] (Figure 2).

### 2.5. Periodontal Phenotype Evaluations

Stern et al. [3] suggested that CBCT should be the standard means for evaluating the amount and positioning of bone regeneration post surgery when measuring the success of SFOT. Therefore, limited-view CBCT images including the mandibular arch (voxel size 0.300 mm) were captured before surgery and six months after surgery. The images from both time points were imported into Dolphin software (version 11.7, Dolphin Imaging & Management Solutions, Chatsworth, CA, USA) and oriented by using the occlusal plane as the horizontal plane. The T2 CBCTs were superimposed on T1 CBCTs by using the voxel-based superimposition method [20,21,22]. The amount and positioning of bone regeneration were visualized at the levels of 3 mm, 6 mm, and 9 mm below the cementoenamel junction (CEJ) of the central incisors.

For the clinical soft tissue assessment, the height of keratinized tissue (KT) was measured from the mucogingival junction to the free gingival margin for each mandibular incisor, canine, and premolar by using a periodontal probe. In addition, the gingival thickness was assessed at 3 mm and 6 mm apical to the gingival margin of each tooth using an endodontic spreader fitted with a rubber stopper and measured on a digital caliper [23,24]. All the measurements were performed before surgery and six months after surgery. Measurement errors were minimized by allowing only one periodontist to perform the measurements, and this periodontist was blinded from the grouping information.

## 3. Results

### 3.1. Adverse Events

**Figure 3:**
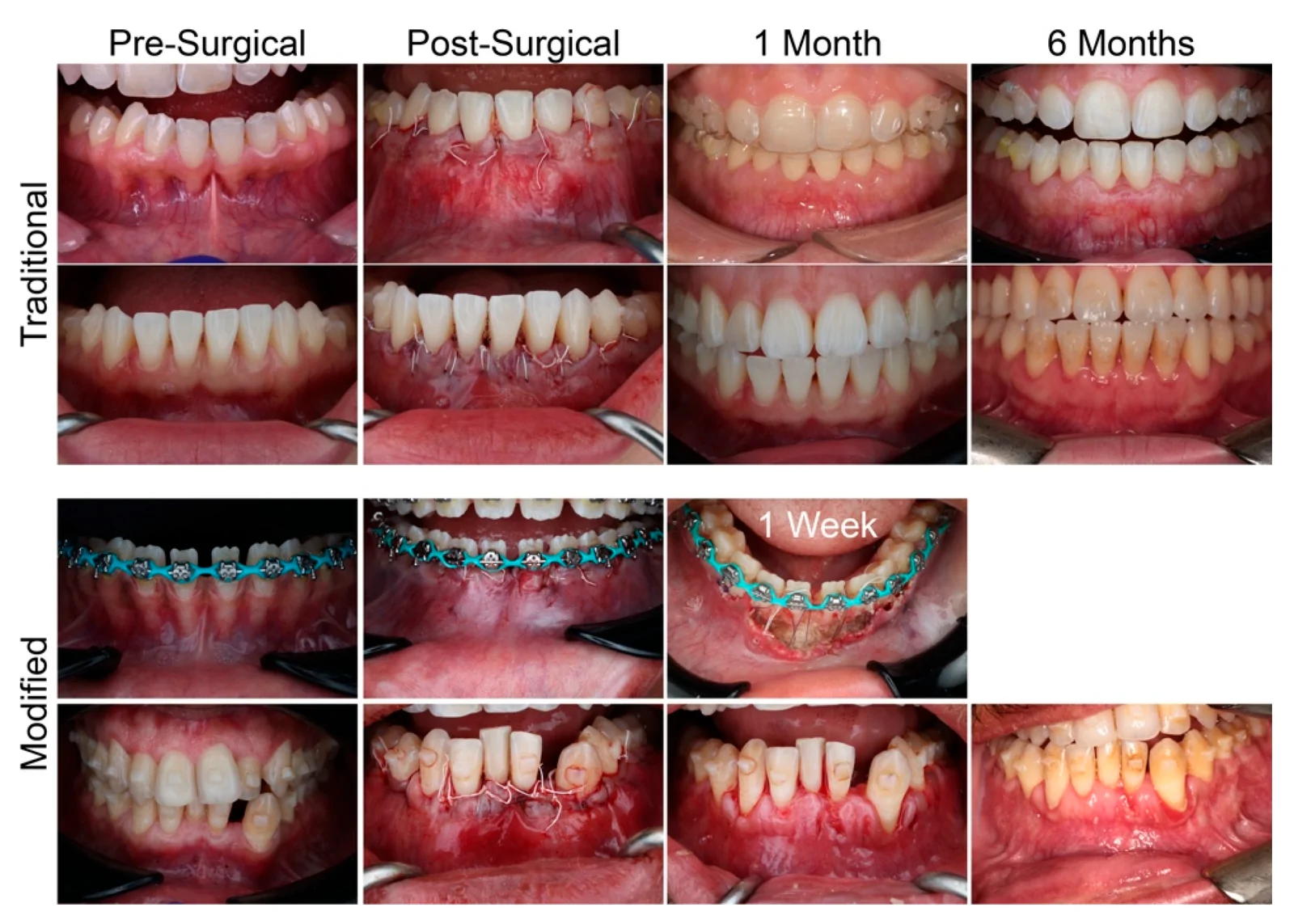
Intraoral photos of the patients at pre-surgical, immediate post-surgical, 1-month follow-up, and 6-month follow-up time points, except the patient who had wound dehiscence one week after surgery.

**Figure 4:**
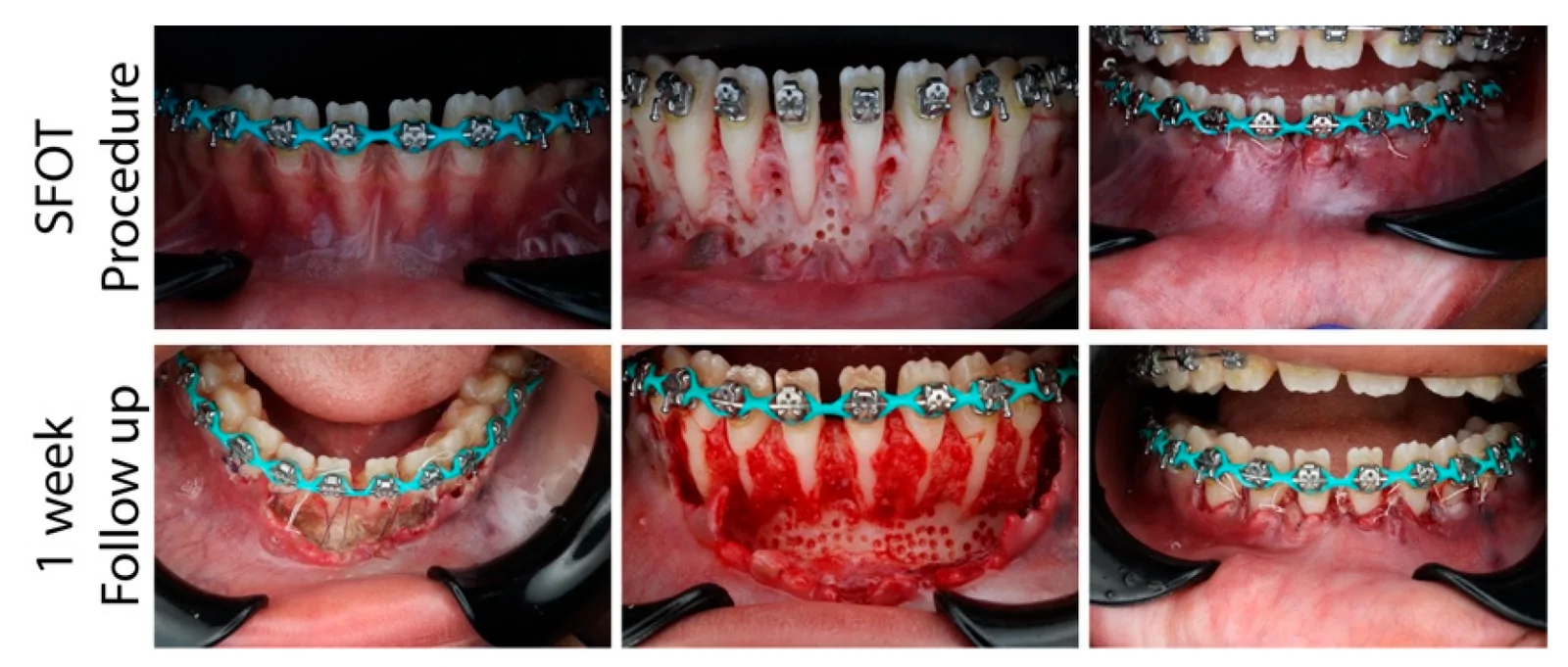
Comparison of the intraoral condition of the patient who had wound dehiscence at the time during the SFOT procedure and at the time during the debridement procedure.

Although primary closure was achieved with all five patients, severe wound dehiscence was observed in one patient from the modified SFOT group one week after the procedure (Figure 3), which led to the removal of the grafting materials (Figure 4). After cleaning out the movable particles, granulation tissue was noticed on the buccal surface of the alveolar bone. For the two patients in the traditional group, the soft tissue appeared to be free of inflammation starting at the 1-month post-surgical follow-up appointment, while for the other patient in the modified group, inflammation was noticed at both 1-month and 6-month post-surgical appointments (Figure 3).

The patients’ discomfort level at the 1-week and 2-week post-surgical time points are presented in Table 2 and Table 3. Overall, the patients in the modified SFOT group appeared to experience more severe discomfort than the patients in the traditional SFOT group one week post surgery (Table 2). More specifically, the two patients in the modified SFOT group reported a 7–10/10 level of pain, discomfort, and swelling. In contrast, the two patients in the traditional SFOT group reported a 2–8/10 score on these three questions. In addition, a distinct difference was observed in the responses to the question “How much bleeding did you experience?” and “How much bruising did you experience?”, with the patients in the traditional SFOT group reporting a score of 0–3/10 and the patients in the modified SFOT group reporting a score of 3–7/10. Two weeks post surgery, the reported discomfort of the patient in the modified SFOT group reduced to a similar level as that of the patients in the traditional SFOT group (Table 3), with the score ranging between 0 and 6, except one patient in the modified SFOT group who still reported an 8/10 difficulty with chewing.

### 3.2. Periodontal Phenotype Evaluations

**Figure 5:**
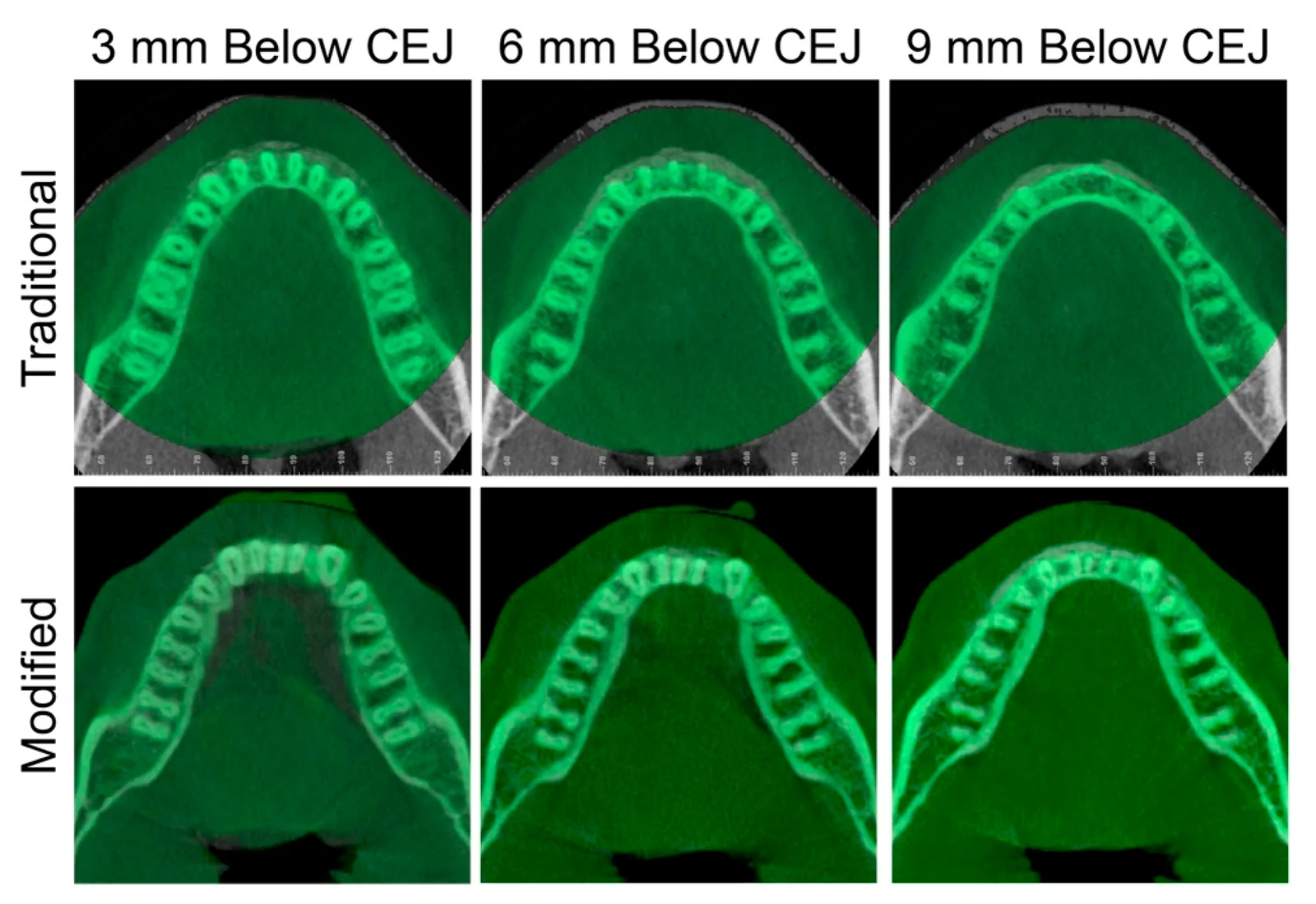
The axial cross-sections of pre- (green) and 6-month post-grafting (white) CBCTs at the levels of 3 mm, 6 mm, and 9 mm below the cementoenamel junction of the mandibular right central incisor.

When evaluating the amount and positioning of bone regeneration on the CBCT images (Figure 5), both subjects (one with traditional SFOT and one with modified SFOT) presented with a significant amount of new bone on the buccal surface of the lower anterior teeth. However, in the subject with traditional SFOT, more bone generation was noticed at the levels of 3 mm and 6 mm below the CEJ, while in the subject with modified SFOT, more bone generation was noticed at the level 9 mm below the CEJ.

**Figure 6:**
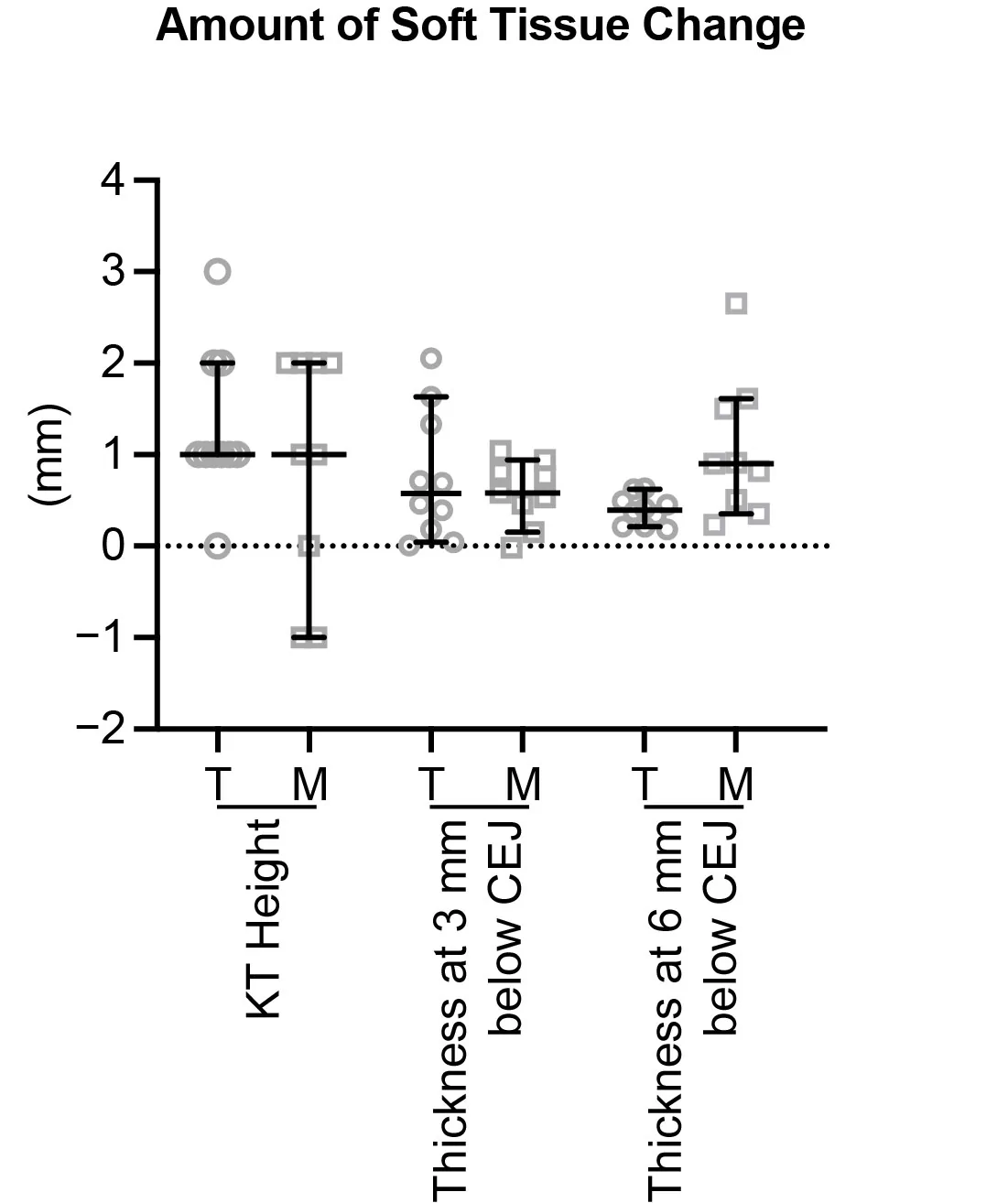
The changes in keratinized tissue (KT) height and gingival thickness of the patients with traditional (T) and modified (M) SFOT. There were ten involved teeth from one patient in the traditional SFOT group and nine involved teeth (due to missing one lower incisor) from one patient in the modified SFOT group. The data are presented as median with a 95% confidence interval.

For the soft tissue measurements (Figure 6), both patients presented with some extent of increase in KT height (traditional: 1.0 mm [0.0 mm, 3.0 mm] vs. modified: 1.0 mm [−1.0 mm, 2.0 mm]). In addition, both patients presented with a significant amount of soft tissue thickening, with a similar amount at the level of 3 mm below the CEJ (traditional: 0.58 mm [0.01 mm, 2.05 mm] vs. modified: 0.58 mm [−0.02 mm, 1.04 mm]). For the soft tissue thickening at the level of 6 mm below the CEJ, the patient with modified SFOT (0.90 mm [0.23 mm, 2.65 mm]) showed a larger amount of thickness increase than the patient with traditional SFOT (0.39 mm [0.18 mm, 0.63 mm]).

## 4. Discussion

In 2015, Wilcko et al. reported that orthodontic treatment alone could reduce the KT height by 0.38 mm at the time point of 1.5 years after orthodontic treatment, while alveolar decortication and augmentation bone grafting with a combination of demineralized freeze-dried bone allograft (DFDBA) and bovine bone xenograft wetted with clindamycin phosphate antibiotic could facilitate an increase in KT height by 1.28 mm [25]. In addition, recently, Chang et al. stated that 6 months after simultaneous labial and lingual augmented corticotomy grafted with reconstituted deproteinized bovine bone (Bio-Oss) and bioabsorbable collagen membranes (Bio-Gide), a 0.66 mm increase in KT height was observed [26]. Meanwhile, Li et al. reported that SFOT with Bio-Oss and Bio-Gide introduced minimum changes in KT height with an increase of 0.46 mm at 1 year after the procedure and 0.07 mm at 3 years after the procedure [27]. In the current case report, a 1.0 mm increase in KT height was observed in both subjects. Thus, SFOT alone could increase the KT height but the addition of collagen matrix did not further enhance this effect. In addition, the difference among the studies may be due to the method of measurements (as Wilcko et al.’s study used two-dimensional intraoral photographs, whereas Chang et al.’s and Li et al.’s studies performed measurements using intraoral scans, and the current study performed direct intraoral measurements), the direction of orthodontic tooth movement, the observation time point, and the types of grafting materials. Further studies are warranted to explore the effects of different bone grafting materials on KT height changes.

In regard to the soft tissue thickness change, a 0.58 mm increase was observed at the level of 3 mm below the CEJ in both subjects in the current study. This amount of soft tissue thickness increase is also similar to the number reported by Chang et al. (0.44 mm at the 6-month timepoint) [26] and Li et al. (0.517 mm at the five-year timepoint) [27]. However, it is worth noting that the current study is the only one that performed bone sounding to accurately evaluate the soft tissue thickness. Furthermore, when evaluating at the level of 6 mm below the CEJ, the subject with modified SFOT (0.90 mm) showed significantly more soft tissue thickness increase than the subject with traditional SFOT (0.39 mm). Thus, SFOT alone could increase the soft tissue thickness and the addition of collagen matrix further enhances this effect, especially at a more apical level.

Besides the potential of increasing soft tissue augmentation by adding collagen matrix to SFOT, there are several clinical observations that need further evaluation and investigation. Firstly, the patients who received modified SFOT generally reported more severe post-surgical discomfort than those who received traditional SFOT, even though the patients in the modified group were given methylprednisolone to reduce the swelling. While periosteal releasing incisions completed in the modified SFOT group could contribute to increased post-operative discomfort, the collagen matrix may contribute significantly to the patient-reported discomfort. In fact, approximately a 25% volume gain occurs when wetting the collagen matrix. Thus, although primary closure could be achieved initially, the swelling of the collagen matrix may induce an increase in tension, which subsequently causes more severe discomfort to the patient. This might also be the reason that caused wound dehiscence and procedure failure on one subject in the modified SFOT group. The swelling of the collagen matrix may also compress the bone particles, leading to the apical migration of bone particles as shown on the 6-month post-procedure CBCT. Therefore, to reduce the risk of wound dehiscence, more extensive periosteal releasing incisions should be considered to ensure tension-free flap management over the collagen matrix during initial healing to account for the volume expansion of collagen matrices that may increase pressure to the flap and the underlying bone particles.

Secondly, high inflammatory responses of the gingival tissue were observed in the subject with modified SFOT, even 6 months after the procedure. It is worth noting that Fibro-Gide^®^ could increase the expression level of MMP-2, a matrix metalloproteinase that is secreted by preinflammatory cells during inflammation, in human fibroblasts in vitro [28]. In addition, in the management of palatal defects in humans, the Fibro-Gide^®^ group showed slower wound closure and more prolonged presentation of nucleated cells than the control and Mucograft^®^ groups. Thus, although a significant amount of newly generated bone was detected on the CBCT, whether the inflammatory responses associated with Fibro-Gide^®^ could interfere with the bone particle resorption, and how to control the inflammatory responses, need further investigations.

An observation to note is the expectation of true regeneration when using particulate grafting on avascular root surfaces, especially in thin-periodontal phenotype patients presenting with significant root dehiscence extending near the apices. As described by Mandelaris et al. [29], this process is more accurately characterized as bone augmentation rather than true regeneration, given the absence of native alveolar bone in areas of fenestration or dehiscence. On the other hand, blood supply from the periosteum plays a critical role in periodontal regeneration [30]. Contemporary evidence highlights that successful wound healing is highly dependent on vascularization and the availability of progenitor cell sources, particularly from the periodontal ligament and periosteum. In this context, the periosteum functions as a biologically active, vascularized tissue that may enhance bone formation compared to membrane-based approaches [31]. Although both subjects in the current study showed a significant amount of increase in buccal alveolar ridge thickness when evaluating the CBCT images, whether the radiopaque area is filled with newly generated bone or just residual bone particles cannot be concluded without histological analysis. The presence of a denuded or avascular root surface does not, by itself, make periodontal regenerative or graft-supported wound healing biologically implausible. Guided tissue regeneration has been described not only for intrabony and furcation defects, but also as a regenerative approach for selected gingival recession/root coverage defects, including more challenging Miller Class III recession defects. In such situations, wound healing is not dependent on vascularity from the root surface itself, but rather on biologic support from the adjacent periodontal ligament, alveolar bone, periosteal/supraperiosteal circulation, and the overlying flap [32].

Lastly, recent studies demonstrated that the depth of corticotomies could impact the orthodontic tooth movement [33,34,35], and higher amounts of osteoclastic activity and new collagen formation were observed when the penetrations were deep [35]. Whether the depth of corticotomies could affect the outcome of SFOT needs further evaluation.

Several limitations of the present study should be considered when interpreting the findings. Firstly, although three subjects were initially enrolled in each treatment group, only one subject per group completed all planned follow-up visits and data collection. As a result, it remains unclear whether the observed differences were primarily attributable to the grafting materials and surgical protocols or to individual patient-related factors, such as age, sex, ethnicity, baseline periodontal phenotype, healing response, and biologic variability. Future clinical trials with adequate sample sizes and controlled study designs are necessary to better determine the true effect of each grafting approach. Secondly, the final evaluation in the present study was performed 6 months after grafting, while comprehensive orthodontic treatment continued beyond this postoperative time point. Although initial leveling and alignment were achieved within 6 months, the periodontal phenotype may continue to change throughout active orthodontic tooth movement and into the retention phase. Therefore, the long-term stability and clinical significance of periodontal phenotype modification should be further evaluated at the completion of orthodontic treatment and during extended follow-up periods, including 5- to 10-year post-treatment assessments.

## 5. Conclusions

This case series highlights the potential of a modified SFOT grafting protocol to achieve simultaneous soft tissue augmentation and enhancement of alveolar bone volume through placement of a collagen matrix between the particulate bone graft and periodontal flap. However, notable postoperative complications, including significant patient discomfort and large wound dehiscence, were observed. These findings suggest that additional refinement of the surgical technique may be necessary to improve patient comfort, enhance flap stability, and reduce the risk of wound-healing complications.

## Figures and Tables

**Table 1:** The experimental design of follow-up appointments, as well as the status of data collection of the current study.

Visit	Data Collection (Provider’s Initial)	Traditional SFOT #1	Traditional SFOT #2	Traditional SFOT #3	Modified SFOT #1	Modified SFOT #2	Modified SFOT #3
Screening	Consent (J.P.)	✔	✔	✔	✔	✔	✔
	Photos (J.P.)	✔	✔	✔	✔	✔	✔
	CBCT (J.P.)	✔	✔	✔	✔	✔	✔
Surgery	Randomization (Y.-C.C.)	✔	✔	✔	✔	✔	✔
	Probing (T.-J.W.)	✔	✔	✔	-	✔	✔
	Surgery (J.P.)	✔	✔	✔	-	✔	✔
1 week follow up	Photos (J.P.)	✔	✔	-	-	✔	✔
	Questionnaire (J.P.)	✔	✔	-	-	✔	✔
	Adverse Event Assessment (J.P.)	✔	✔	-	-	Wound dehiscence	✔
2 week follow up	Photos (J.P.)	✔	✔	-	-	✔	✔
	Questionnaire (J.P.)	✔	✔	-	-	✔	✔
	Adverse Event Assessment (J.P.)	✔	✔	-	-	✔	✔
	suture removal (J.P.)	✔	✔	-	-	✔	✔
1 month follow up	Photos (J.P.)	✔	✔	-	-	-	✔
	Adverse Event Assessment (J.P.)	✔	✔	-	-	-	✔
6 month follow up	Photos (J.P.)	✔	✔	-	-	-	✔
	Adverse Event Assessment (J.P.)	✔	✔	-	-	-	✔
	Probing (T.-J.W.)	✔	-	-	-	-	✔
	CBCT (J.P.)	✔	-	-	-		✔

**Table 2:** Responses to the questionnaire at 1-week post-op. Each column represents the responses from one subject.

	Traditional		Modified	
	Traditional SFOT #1	Traditional SFOT #2	Modified SFOT #2	Modified SFOT #3
How much pain did you experience?	2	6	7	10
How much discomfort did you experience?	3	8	7	10
How much swelling did you experience?	2	8	7	10
How much difficulty did you have with chewing?	2	9	8	9
How much difficulty did you have with swallowing?	2	3	5	3
How much difficulty did you have with limitations to jaw movement?	3	7	4	8
How much bleeding did you experience?	3	0	5	7
How much bruising did you experience?	1	0	3	5
If feverish at any point, how severe was your experience?	0	0	0	0
If difficulty breathing at any point, how severe was your experience?	0	0	0	0

**Table 3:** Responses to the questionnaire at 2 weeks post-op. Each column represents the responses from one subject.

	Traditional		Modified	
	Traditional SFOT #1	Traditional SFOT #2	Modified SFOT #2	Modified SFOT #3
How much pain did you experience?	2	5	4	2
How much discomfort did you experience?	2	6	4	6
How much swelling did you experience?	1	4	3	0
How much difficulty did you have with chewing?	1	5	4	8
How much difficulty did you have with swallowing?	0	0	4	0
How much difficulty did you have with limitations to jaw movement?	0	3	4	1
How much bleeding did you experience?	0	1	1	0
How much bruising did you experience?	0	0	0	1
If feverish at any point, how severe was your experience?	0	0	0	0
If difficulty breathing at any point, how severe was your experience?	0	0	0	0

## Data Availability

The original contributions presented in this study are included in the article. Further inquiries can be directed to the corresponding authors.
